# A topographical and physiological exploration of C-tactile afferents and their response to menthol and histamine

**DOI:** 10.1152/jn.00310.2021

**Published:** 2022-01-12

**Authors:** Line S. Löken, Helena Backlund Wasling, Håkan Olausson, Francis McGlone, Johan Wessberg

**Affiliations:** ^1^Department of Physiology, Institute of Neuroscience and Physiology, University of Gothenburg, Gothenburg, Sweden; ^2^Department of Biomedical and Clinical Sciences, Center for Social and Affective Neuroscience, Linköping University, Linköping, Sweden; ^3^Research Centre for Brain & Behaviour, School of Natural Sciences and Psychology, Liverpool John Moores University, Liverpool, United Kingdom

**Keywords:** affective touch, CT afferent, histamine, menthol, peroneal nerve

## Abstract

Unmyelinated tactile (C-tactile or CT) afferents are abundant in arm hairy skin and have been suggested to signal features of social affective touch. Here, we recorded from unmyelinated low-threshold mechanosensitive afferents in the peroneal and radial nerves. The most distal receptive fields were located on the proximal phalanx of the third finger for the superficial branch of the radial nerve and near the lateral malleolus for the peroneal nerve. We found that the physiological properties with regard to conduction velocity and mechanical threshold, as well as their tuning to brush velocity, were similar in CT units across the antebrachial (*n* = 27), radial (*n* = 8), and peroneal (*n* = 4) nerves. Moreover, we found that although CT afferents are readily found during microneurography of the arm nerves, they appear to be much more sparse in the lower leg compared with C-nociceptors. We continued to explore CT afferents with regard to their chemical sensitivity and found that they could not be activated by topical application to their receptive field of either the cooling agent menthol or the pruritogen histamine. In light of previous studies showing the combined effects that temperature and mechanical stimuli have on these neurons, these findings add to the growing body of research suggesting that CT afferents constitute a unique class of sensory afferents with highly specialized mechanisms for transducing gentle touch.

**NEW & NOTEWORHY** Unmyelinated tactile (CT) afferents are abundant in arm hairy skin and are thought to signal features of social affective touch. We show that CTs are also present but are relatively sparse in the lower leg compared with C-nociceptors. CTs display similar physiological properties across the arm and leg nerves. Furthermore, CT afferents do not respond to the cooling agent menthol or the pruritogen histamine, and their mechanical response properties are not altered by these chemicals.

## Graphical abstract

**Figure:**
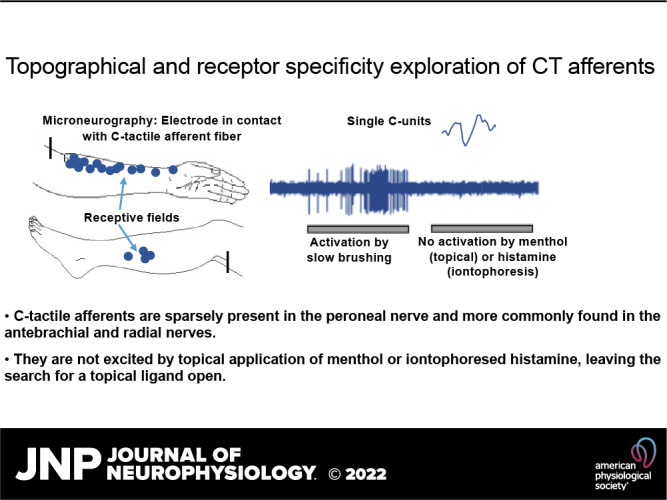

## INTRODUCTION

Since the discovery that the skin of humans is innervated with unmyelinated tactile afferents (C-tactile or CT) that convey social and emotional aspects of touch (1–4), extensive explorations of the physiology and function of these afferents have been made (5–8). Despite this, aspects of the properties of CT afferents as well as their prevalence and density in different areas of the human body have remained elusive. The earliest observations of the presence of CTs in human skin were made by Johansson et al. (9) during microneurography recordings in the supra- and infraorbital nerves, and 2 years later, Nordin (10) described the physiological properties of CTs in these nerves in much finer detail. The finding that CT afferents showed a vigorous response to stroking with a soft cotton swab was particularly intriguing, as C-fibers were generally thought to convey temperature, itch, and nociception. Because such a large body of microneurography studies had been performed on the nerves of the arms and legs (11–13), the data presented by Nordin were initially thought to be specific for the trigeminal nerve. This turned out to not be true. Shortly thereafter, during microneurographic registrations from the lateral and dorsal antebrachial cutaneous nerves, CT afferents were found to be densely represented in the nerves of the arm as well (4, 14). In addition, CT afferents were later also observed in the lateral cutaneous femoral nerve, innervating the thigh (15).

The physiological characterizations of CT afferents have shown that they display characteristics such as fatigue, afterdischarge activity, intermediate adaptation, and sensitivity to mechanical stimuli and cooling stimuli (1, 3, 4, 10, 14, 16). These properties, and in particular their poor temporal coding abilities and high activity during slow stroking movements, led to the proposal that CT afferents constitute a separate pathway mediating affective touch in the body (2, 4). This proposal was ratified by the finding that there is a negative quadratic relationship between brush velocity and mean firing rate, which is mirrored in perceptual ratings of pleasantness in response to the same stimuli (1) and other smooth materials (17). Furthermore, the tuning to slow touch that CT afferents display is strongest at temperatures that mimic skin-to-skin contact (18). CT afferents are clearly separable from C-mechanonociceptors by having a mechanical threshold of 2.5 mN or lower (4) and are insensitive to heat (4, 10). Analysis of the responsiveness to cooling and combined mechanical and temperature stimuli has revealed that they do not appear to signal cooling alone but that the combination of cooling and mechanical stimulation may alter their firing properties (10, 16).

Despite these extensive investigations, fundamental questions remain regarding the distribution of CT afferents in the more distal parts of the limbs and their sensitivity to topically or transdermally delivered chemical compounds. Recent studies have shed further light on the gene expression that may be underlying specific transduction mechanisms in mouse C low-threshold mechanoreceptors (CLTMRs, the mouse equivalent of CTs) (19). It is therefore of increasing interest to establish the degree to which rodent CLTMRs and human CTs have shared characteristics, as this opens up possibilities for translational studies. Studies in mice describe CLTMRs as innervating specific types of hair follicles, and this may well be similar in human skin. However, it is also known that the human epidermis is densely innervated by free nerve endings, and some of these may be CTs, rendering them as an easy target for topical or iontophoretically applied ligands (20). We therefore first set out to investigate the presence of CT afferents in different cutaneous nerves and how their respective physiological properties compare across skin sites. Then, as the chemical sensitivity of CT afferents is largely unexplored, we examined the responsiveness of CT afferents to histamine, a well-known pruritogen (21), and menthol, a ligand for the TRPM8 cold-sensitive receptor (22–25). It should be stated at the outset that our findings on chemical sensitivity are not final but may serve as a foundation for further exploration.

## METHODS

### Participants

The participants were recruited through advertisements posted in the university and mainly included students in the medical department. Forty-five participants were included in the study and were required to be healthy, with no neurological illness. The mean age was 24 yr (range = 20–31), and 13 were male. Written, informed consent was acquired before commencing experiments, and financial compensation was given for their time. The University of Gothenburg Ethics Committee approved the experimental protocol that was performed in accordance with the Declaration of Helsinki.

### Experimental Procedure

Using the microneurography technique (26), we recorded from single CT afferents in the antebrachial and radial nerves that innervate the arm and from CT and C-nociceptors in the peroneal nerve that innervate the leg. Participants were seated comfortably in an adjustable dental chair with their left arm or leg supported by a vacuum airbag for stability. A custom-made preamplifier (Department of Physiology, Umeå University) and a silver-plated ground plate were attached to the participant’s forearm or leg.

### Search Procedure

We used an electrical search procedure to locate the nerves. The electrode was inserted below the fibular head for the peroneal nerve, at the level of the elbow for the antebrachial nerve, and at the dorsal aspect of the forearm for the radial nerve. The skin was palpated to find the ideal place for insertion of the stimulating and recording electrodes, and an uninsulated reference electrode was inserted ∼5 cm from this site. The stimulating electrode was uninsulated (35 or 50 mm length, 200 µm shaft diameter, ∼5 µm tip diameter; FHC, Bowdoin, ME) and was used to deliver 200µs square, negative, 1-Hz pulses at low current until the participant reported paresthesia in the innervation area. Once an ideal electrode position was obtained, the depth and angle of the electrode were noted, and the electrode was subsequently withdrawn slightly from its proximity to the nerve. An insulated tungsten electrode (FHC, UNA35FNM) was then inserted at the noted depth and angle distal to the search electrode. Recordings were made from single afferents. When the tip had attained an intrafascicular position, the experimenter searched for single units by lightly stroking with fingertips over the skin on the surface of the innervation area and making minimal adjustments of the electrode position. Single units that were identified as unmyelinated (by negative spike deflection and latency), responded to soft brush stroking, and had amplitudes distinct from the noise were further studied. The nerve signal was recorded at 12.8 kHz with a passive band-pass filter set to 0.2–4.0 kHz and stored on a PC using the ZOOM/SC system developed at the Department of Physiology, Umeå University, Sweden. Recorded nerve impulses (spikes) were inspected offline on an expanded time scale using in-house software implemented in MATLAB (The MathWorks, Natick, MA) and were accepted for subsequent analyses only if they could be validated as originating from a single afferent.

### Unit Identification

We identified CT afferents according to the criteria set in previous studies (3, 4, 14). First, conduction velocity was measured from the response latency to mechanical tap stimulation using a handheld strain gauge device. Distinct taps with a blunt probe were delivered toward the most sensitive spot within the receptive field, and the minimal latency from indentation to unit response was used to estimate conduction velocity. Myelinated afferents (conduction velocity of >2 m/s) were also studied but are not further described here. Thresholds to mechanical stimuli were assessed using von Frey monofilaments and defined as the weakest stimulation force that the unit consistently responded to. Unmyelinated afferents (conduction velocity of <2 m/s) were classified as CT afferents if they displayed a clear response (several impulses) to low mechanical threshold monofilament bristles below 2.5 mN and vigorous response to brush stroking. Unmyelinated afferents were classified as nociceptors if they displayed a high mechanical threshold to monofilament bristles (>5 mN) and no response to brush stroking. For all CT afferents, conduction velocity was calculated based on response to distinct taps with a strain gauge. Conduction velocity in nociceptors was measured in the same fashion as well as with electrical stimulation, which confirmed the accuracy of the strain gauge measures.

### Experiment

#### Mechanical stimuli.

Units were initially explored with a number of handheld mechanical stimuli such as gentle touch by finger stroking across the receptive field, wooden sticks, and a handheld soft watercolor brush stroked across the receptive field. The presence of afterdischarge, which may occur upon initial stimulation, was noted in the protocol. For some units, we also tested the response to long-lasting indentation with a suprathreshold von Frey filament (see results for number of units tested in this way). The timing of these handheld stimuli was indicated with a foot pedal.

After identification of a CT unit, we applied one or more of the following tests.

##### Robotic tactile stimulator.

Stimulation was made as in the study by Löken et al. (1). In short, we used an artist’s flat, soft watercolor brush made of fine, smooth goat’s hair (Vang size 18, type 43718, Oskar Vangerow, Ottobrunn, Germany). The bristles were 22 mm long, and the width of the brush was 20 mm. Following unit identification, brush stroking was applied by means of a custom-built robotic tactile stimulator (RTS) (Dancer Design, Saint Helens, UK) that produced brush stroking with velocities of 0.1, 0.3, 1, 3, 10, or 30 cm/s. The brush was moved over the skin in a rotary fashion by a DC motor (Maxon Motor AG, Sachseln, Switzerland) fitted with a reduction drive and position encoder. A 6-axis force/torque transducer (ATI Industrial Automation, Apex, NC) was mounted between the shaft of the DC motor assembly and the hub, which held a probe and brush. The DC motor and transducer assembly was mounted on a linear drive, driven by a step motor (Parker Hannifin Corp., Rohnert Park, CA). Both the DC and step motors were under computer control. Stimuli of different velocities were applied in randomized order with an interstimulus interval of 30 s for CT afferents (to avoid fatigue). In experiments where histamine or menthol was applied, we used 10-s interstimulus intervals when testing responsiveness to brush due to the time constraints inherent in these long recording protocols.

##### Menthol.

After localization and characterization of CT units (see *Search Procedure*), recording commenced and the receptive field was first stimulated with a handheld brush. Subsequently, we recorded the normal response to brushing using the RTS. The direction of brush strokes was set from proximal to distal and with normal force at calibration 0.2 N. Slow and fast brush strokes (1 and 10 cm/s) were delivered at intervals of 10 s. This protocol was repeated four times followed by recording and application of ethanol for 5 min. Subsequently, the brush stimulation was repeated as mentioned earlier, after which a pad of menthol solution (30% in ethanol) was applied on the skin for 5 min. We asked the participants to report whether there was any sensation of cooling and then repeated the mechanical stimulation protocol again. The recording continued while asking the participant about the sensation of cooling at intervals of 60 s during 5 min. The skin was then cleaned with ethanol and water. Nerve recordings were maintained throughout the experiment until well after the skin had been cleaned.

##### Histamine.

After localization and characterization of CT units as mentioned earlier, recording commenced, and the receptive field was stimulated with a handheld brush. We alternated application of fast and slow strokes (1 and 10 cm/s) with 10-s intervals. The incidence of brush strokes was noted in the recording, and the procedure was repeated three times. We then recorded baseline with no stimulus for 2 min. Subsequently, a drop of saline was applied to the receptive field, and following, this we applied iontophoresis, 100 µA (= 1 mA, 10%), for 10 s. After 2 min, the saline was wiped off and brush strokes were again applied, as mentioned earlier. After these baseline measures, we applied a drop of histamine (1%) and iontophoresis commenced, 100 µA (= 1 mA, 10%), for 10 s. We waited 2 min and noted the development of flare and wheal. If flare and wheal were missing, the iontophoresis procedure was repeated, otherwise the receptive field was wiped with saline and fast and slow brushing was repeated three times followed by a 2-min recording without stimulation. We noted the development of itch, flare and wheal, the time of their peak, and subsequent attenuation throughout this procedure. In conjunction with noting the development and attenuation of the flare and wheal, participants were asked to report the qualitative sensation and specifically whether there was any sensation of itch. We documented the participant’s verbal reports starting at 2 min after histamine iontophoresis and continuing at 3-min intervals for 20 min following the histamine iontophoresis.

### Analysis

The mean firing rate was calculated from the mean of the shortest interspike intervals. Firing rates were then reported as mean and standard error of mean (SE) for each individual unit. Parametric tests were not used where sample size was low. Regression analysis for curve fit was done by transforming velocity, the independent variable, to log10 values. Calculations were done in MATLAB and SPSS.

## RESULTS

CT afferents (lateral antebrachial nerve: *n* = 27/32 experiments, radialis: *n* = 8/9 experiments, peroneus: *n* = 4/17 experiments) were identified by a low mechanical threshold to monofilament bristles (lateral antebrachial nerve: *n* = 27, mean threshold = 0.85 mN, range = 0.04–2.5 mN; radial nerve: *n* = 8, median threshold = 0.68 mN, range = 0.27–2.5 mN; peroneal nerve: *n* = 4, median threshold = 1.6 mN, range = 0.7–1.6 mN), slow conduction velocity (lateral antebrachial median = 0.9 m/s, radialis median = 0.98 m/s, peroneus median = 1 m/s, range = 0.9–1.1 m/s), and vigorous response to brush stroking. Sixteen of the units in the sample recorded from the lateral antebrachial nerve have been described previously with respect to their response to brush velocity (1), and one unit from the radial nerve was included in a previous publication (34). The remaining units, including those where menthol or histamine was applied, are not previously reported. The most distal receptive fields were located on the dorsal aspect of the proximal phalanx of the third finger for the superficial branch of the radial nerve and ∼6 cm proximal to the lateral malleolus for the peroneal nerve. C-nociceptors were identified by high-threshold >5 mN indentation with monofilaments, and none of the units responded to a soft brush stroke. One unit responded with a few impulses to finger stroking of the receptive field (*n* = 20, threshold range = 5.4–59 mN, mean = 27 mN, mean conduction velocity = 0.9 m/s).

### Distribution of CT Units across the Skin

**Figure 1.**
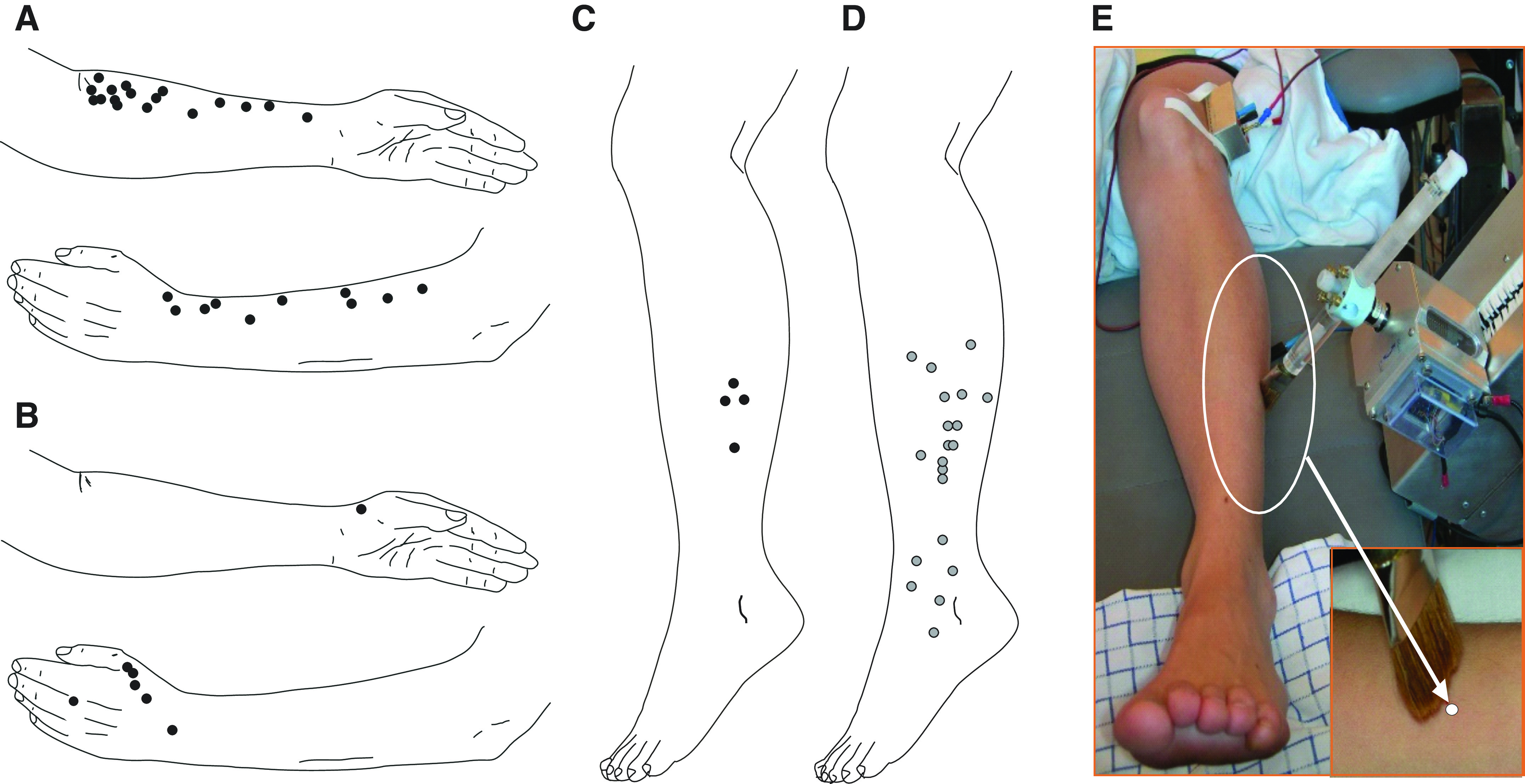
Distribution of C-tactile (CT) units across the skin. Dots mark the location of all recorded CT units on arms (*A* and *B*) and legs (*C*) and nociceptors are marked in *D*. The setup and brush stimulus for the peroneal nerve recordings is shown in *E*. The recordings included CT units in the lateral antebrachial nerve (*n* = 27/32 experiments), radialis (*n* = 8/9 experiments), peroneus (*n* = 4/17), and nociceptor units in peroneus (*n* = 20/17 experiments).

We performed an extensive search for CT units in the peroneal nerve and obtained an intrafascicular position for stable recording in 17 experiments. In the peroneal nerve, background sympathetic activity was commonly recorded, making the identification of C-units more challenging, which in contrast is unusual when recording from the radial or lateral antebrachial nerves. We used a similar search technique as for the nerves of the arm (i.e., stroking the skin while slowly adjusting the recording electrode). For this nerve, we also routinely applied pinching to the skin to identify high-threshold C-fibers. The purpose was to assess whether C-afferent identification was generally difficult in the peroneal nerve, due to background activity, or whether the difficulty was specific to CT afferents. In this sample, we found five times more high-threshold C-fibers (*n* = 20) compared with low-threshold C-afferents (*n* = 4). In relative terms, the detection of a CT unit in the peroneal nerve was less common than for the nerves of the arm (4 units from 17 peroneal experiments vs. 35 units from 41 experiments for the forearm nerves). Pinching was not routinely applied in experiments on the arm nerves. We marked the location of all recorded CT units across the arms and legs for the lateral antebrachial, radial, and peroneal nerves as well as C-nociceptors in the peroneal nerve (see Fig. 1, *A*–*D*). To explore the response to brush stroking in the different nerves, we used the RTS. The setup for brush stroking across the receptive field of units in the lower leg is shown in Fig. 1*E*.

### Mechanical Response Properties in Units across Nerves

**Figure 2.**
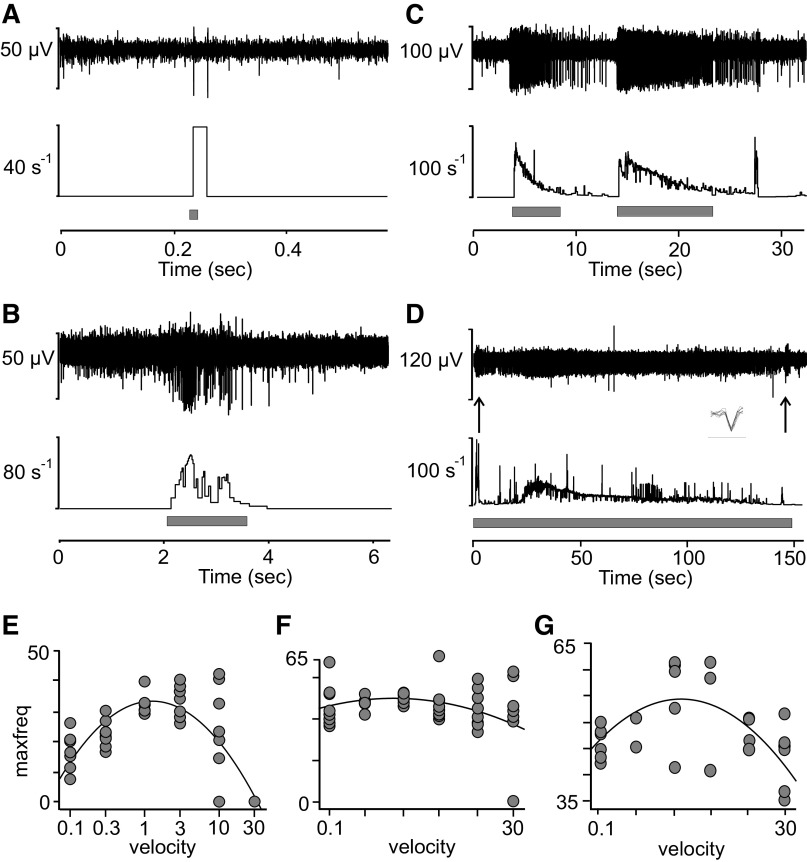
Mechanical (brush) response properties in units across nerves. Spikes in response to fast (*A*) and slow (*B*) brush in peroneal nerve unit. *C*: vigorous afterdischarge in C-tactile (CT) afferent in response to brush stimulation. The figure shows several consecutive brush strokes. *D*: delayed acceleration of impulse response to sustained monofilament indentation (45 mN) in peroneal nerve unit. The initial few seconds of adaptation were followed by a period of low activity for 20 s, after which firing markedly increased again for about 90 s. Note that individual spikes are not discernable and almost completely covers signal background. *Inset*: individual spikes superimposed (bar below spikes denotes time 1 ms). Arrows denote on and off stimulation where an increased firing is seen as the indentation stops. *E*–*G*: single-unit examples of firing across units in the different nerves in response to brush stroking velocity (0.1–30 cm/s) and fitted quadratic curve: lateral antebrachial nerve (*E*), radial nerve (*F*), and peroneal nerve (*G*). Each dot in *E–G* represents a single brush stroke.

**Figure A1.**
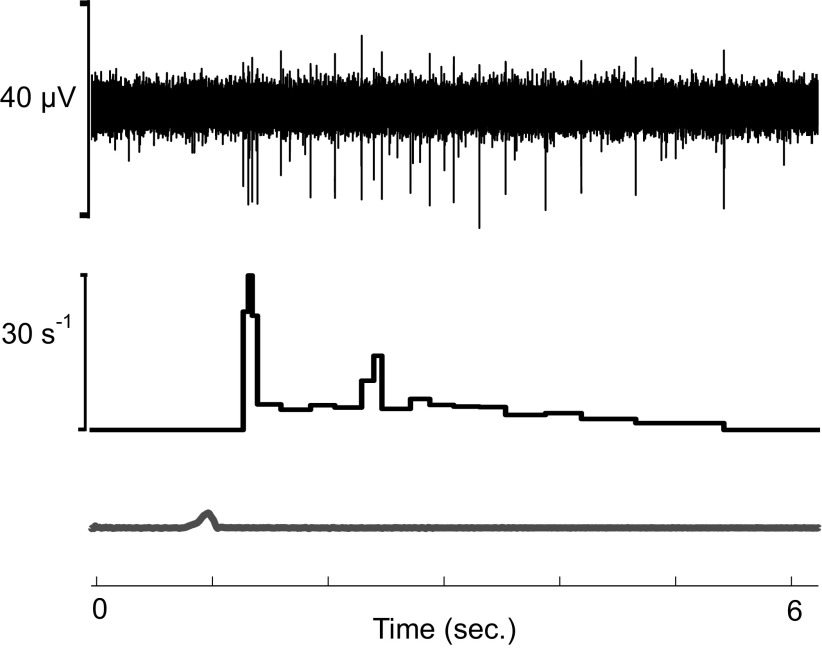
Example of afterdischarge in a C-unit with high mechanical threshold (3 g) in response to a sharp mechanical probe. *Bottom*: timing of the probe stimulation and the firing continues for several seconds after the probe is removed.

We compared basic properties of C-tactile afferents across the different nerves. We have previously shown the response to brush stroking at velocities of 0.1–30 cm/s in the lateral antebrachial nerve (1). Here, we explored two of the four peroneal nerve units in the same fashion, and they showed similar response properties to brushing as described for CT units of the arm (Fig. 2, *A* and *B*, shows single stroke data from a peroneal nerve unit). A fast brush stroke (30 cm/s, Fig. 2*A*) typically only evokes a couple of impulses in these units, whereas a slow brush stroke (3 cm/s, Fig. 2*B*) typically evokes a vigorous response. None of the four CT units in the peroneal nerve exhibited afterdischarge during our recordings. Four out of 20 nociceptors recorded from the peroneal nerve displayed afterdischarge in response to a mechanical tap by the strain gauge upon initial exploration (Fig. A1). Afterdischarge is a relatively common feature in CT units of the arm and appeared in 11 out of 35 units. A clear example is shown in Fig. 2*C* where the unit, recorded from the lateral antebrachial cutaneous nerve, repeatedly fired with an extensive tail of impulses for several seconds after the stimulus had left contact with the skin. The subject could not report of any particular sensation in conjunction with this phenomenon, and its functional relevance remains unknown. We also tested delayed acceleration in all CT units of the peroneal nerve. One afferent, recorded from the peroneal nerve, showed a delayed acceleration of impulse response to sustained monofilament indentation (45 mN) (Fig. 2*D*). The initial few seconds of adaptation were followed by a period of low activity for 20 s, after which firing increased markedly again for ∼90 s. This phenomenon, called delayed acceleration, has previously been reported in some CT units in the lateral antebrachial cutaneous nerve (4). In the peroneal nerve, out of three units tested with sustained indentation, one unit showed a delayed acceleration response (Fig. 2*D*). In the arm experiments, we found two units out of seven tested that showed delayed acceleration. Note that the timescale is long in this figure and that individual spikes cover the view almost completely of the background. Individual spikes are superimposed in inset. The tuned response to intermediate brush velocity shown for CT afferents previously was consistent across units in the different nerves. Figure 2, *E*–*G*, shows single-unit examples in response to the full brush stroking protocol (0.1–30 cm/s) in the lateral antebrachial (Fig. 2*E*), radial (Fig. 2*F*), and peroneal (Fig. 2*G*) nerves. Although some variability is present in individual units as a response to the brush, the inverted U-shaped curve (quadratic fit) of firing rate as a function of velocity is highly consistent.

After establishing the prevalence of CT units in these nerves and finding that they display similar mechanical response properties in the lower leg as in the arm, our next outstanding question was to explore their sensitivity to chemical agents. Although we had no direct indication from previous studies that CT afferents, or their mouse homologue CLTMs, are sensitive to chemicals, a formal exploration in humans was lacking. We therefore here chose to evaluate the responses to two well-known neuroexcitatory chemical agents: menthol and histamine.

#### Menthol.

**Figure 3.**
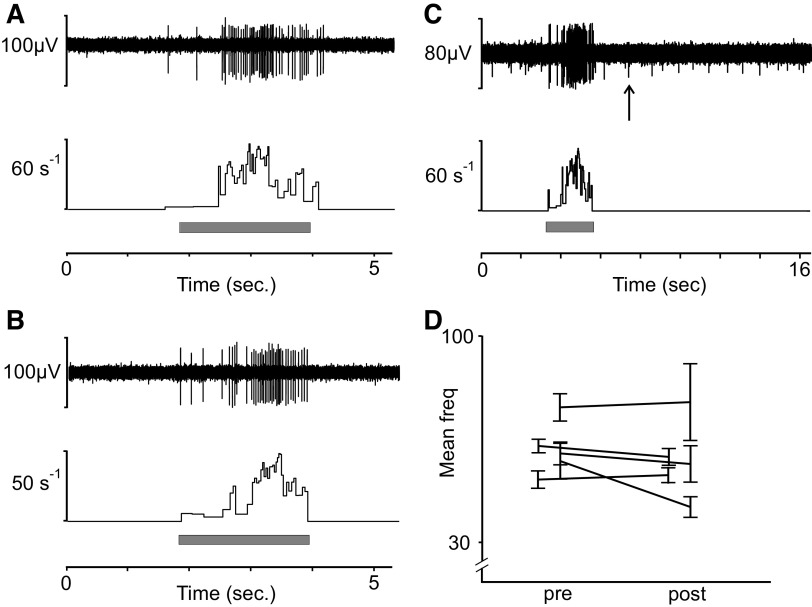
C-tactile (CT) afferent response to menthol. Tactile stimulation with brushing at 1 cm/s of CT afferent before (*A*) and after (*C*) menthol application. *B*: cooling unit in background responding to menthol at time of cooling percept is indicated by arrow. *D*: mean and standard error (SE) of firing rate for individual units before (pre) and after (post) application (mean of all units pre = 61 Hz, SE = ±1.9 Hz and post = 50 Hz, SE = ±2.4 Hz). Menthol was applied in *n* = 8 units. Brushing pre- and post menthol application was recorded in *n* = 5 units.

In a new set of recordings, we returned to the nerves of the arm (lateral antebrachial nerve) where CT units are readily found. Here, we examined the response to menthol application on the receptive field of eight CT units. For all experiments, we initially applied ethanol as a control on the receptive field. We also noted the participant’s perception of cooling throughout the experiment. There was no activity in CT afferents in response to menthol alone. We confirmed the effect of menthol by perceptual reports of cooling sensation and recording from a C-afferent that was active in the background, suggesting sensitivity to cooling (see arrow denoting smaller unit Fig. 3*C*). In several recordings, a background C-unit near the recording electrode, whose activity did not correlate with mechanical stimulation, would appear at latency that matched the perception of cooling sensation. We also analyzed whether the firing frequency in response to brushing was altered by the menthol in five units. There was no indication that the fast (10 cm/s) and slow (1 cm/s) strokes had any different effect, and we therefore pooled the data from these brush velocities. We found no clear indication that menthol modulated the firing rate in response to brush stroking before (Fig. 3*A*) or after (Fig. 3*B*) menthol application. The mean of all unit firing in response to brush was 61 Hz, SE = ±1.9 Hz, before menthol application (pre) and 50 Hz, SE = ±2.4 Hz, after the application (post). The individual unit firing in response to brushing pre- and postmenthol application is visualized in Fig. 3*D*.

#### Histamine.

**Figure 4.**
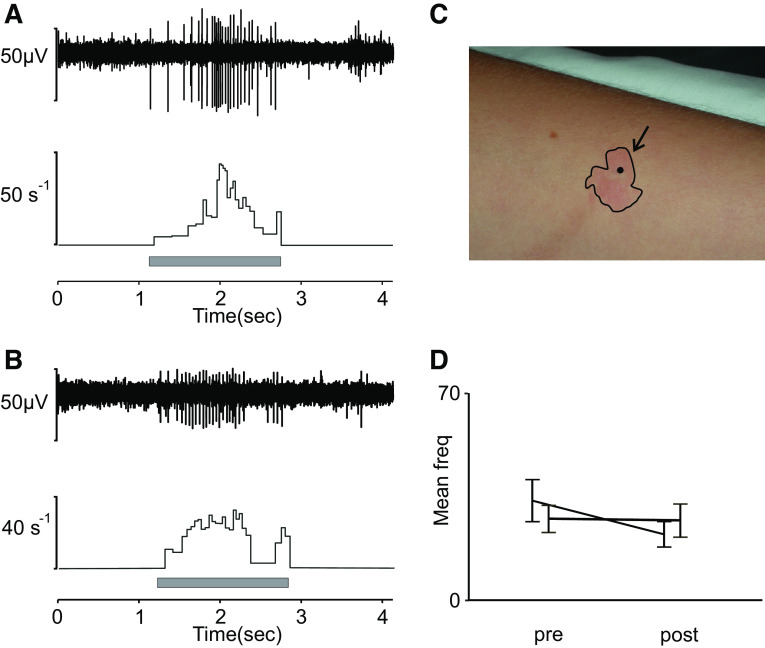
C-tactile (CT) afferent response to histamine. *A*: response to slow brushing (1 cm/s) in CT unit before iontophoresis. *B*: single-unit example of response to the same stimulus after histamine iontophoresis. *C*: wheal and flare to confirm reaction. *D*: mean and standard error (SE) of firing rate for individual units before (pre) and after (post) iontophoresis (mean of both units pre = 28 Hz, SE = ±3.8 Hz and post = 23 Hz, SE = ±3.3 Hz). Histamine was applied in *n* = 5 units. Brushing pre- and post histamine was recorded for *n* = 2 units.

We continued our exploration in another set of experiments, also recorded in the arm nerves (lateral antebrachial and radial nerve). We explored the reaction to histamine iontophoresis in five CT units. CT units were characterized as mentioned earlier, and a positive reaction to the histamine was confirmed by a clear flare, and occasionally wheal, on the skin region surrounding the units after iontophoresis. Figure 4*B* shows a typical flare reaction on the skin surrounding the receptive field of a CT afferent. Iontophoresis of histamine did not evoke any activity in any of the five CT afferents. To further assess the effect of histamine on CT afferents, we tested whether the response to a mechanical stimulus was altered by the application of histamine by comparing the response to brush stroking before and after application. We applied fast (10 cm/s) and slow (1 cm/s) brush strokes over the course of the experiment. In two of these units, we were able to keep a stable recording throughout this extended protocol. Figure 4 shows a typical, vigorous response to slow brushing (1 cm/s) in one of these units that is similar before (Fig. 4*A*) and after (Fig. 4*B*) histamine iontophoresis. There was no indication that the fast (10 cm/s) and slow (1 cm/s) strokes had any different effect, and we therefore pooled the data from these brush velocities. Figure 4*C* illustrates an example of a flare response to the histamine, and Fig. 4*D* illustrates the mean and standard error of firing rate in response to brushing for each unit before and after iontophoresis [mean of pooled units before (pre) = 28 Hz, SE = ±3.8 Hz and after (post) = 23 Hz, SE = ±3.3 Hz]. We found no indication from these units that mechanical responses were altered by the histamine.

We also documented the participant’s perception starting at 2 min after histamine iontophoresis and continuing at 3-min intervals for 20 min following the histamine iontophoresis. Although the concentration used evoked a clear reaction in the skin, and typically evokes itch sensation (27, 28), the participants did not here report any clear sensation in relation to the histamine 3 min after application. The size of the flares was measured and ranged from 15 to 22 mm at their maximum. In the example shown in Fig. 4*B*, the flare had a diameter of 13 mm at 3 min after iontophoresis and extended to maximum 15 mm.

## DISCUSSION

There is overwhelming evidence that CT afferents are present across the hairy skin of mammals, but a comprehensive account of these afferents’ presence across different skin sites in human skin has been lacking. We here describe the presence of CT afferents in the peroneal nerve and compare their incidence in microneurographic recordings with that of the lateral antebrachial and radial nerves. The most distal receptive fields were located on the proximal phalanx of the third finger for the superficial branch of the radial nerve and approximately 6 cm proximal to the lateral malleolus for the peroneal nerve. CT units also responded in a similar fashion across the antebrachial, radial, and peroneal nerves with regard to their physiological properties such as conduction velocity, mechanical threshold, and their tuning to brush velocity.

### Prevalence of CT Afferents across Nerves

Despite numerous microneurographic recordings from the peroneal nerve, CTs have not previously been reported in this nerve. The reason is probably in part because most microneurography studies of the peroneal nerve have relied on distinguishing C-mechanoreceptive afferents based on intrinsic properties of axonal conduction latency changes in response to repetitive electrical stimulation or combined with natural stimuli, a method known as the “marking technique” (29–31). Originally well validated and used for C-mechanonociceptors (CMs), it was only recently adapted to detect nonnociceptive CTs (28, 32). We did an extensive experimental series where we actively searched for and identified four CT afferents in the lower leg. Considering the number of experiments (17) where we actively searched for CT units in the peroneal nerve, the number of CT units identified was relatively low, compared with that in experiments for the arm. An important factor that influences the incidence of identifying different afferents in single-unit recordings is the search technique. Our search technique for low-threshold mechanoreceptors is light stroking of the skin. Using this technique, identifying CT afferents is much more common in the nerves of the arm, where we are as likely to identify CT afferents as we are to record slowly adapting type 1 units (SA1). This prevalence of CT afferents relative to SA1 has been consistent across different studies (1, 3, 4, 16). Part of the difficulty in identifying CT afferents in experiments of the peroneal nerve was that sympathetic background activity (33) was more common than in the skin nerve branches of the arm. Although efferent activity was readily identified, we decided to actively compare the incidence of CT afferents with high-threshold C-fibers responding to pinching the skin. When applying a natural stimulation search technique, we found that CT afferents may indeed be sparser than CMs in the lower leg. Actively stroking or pinching the skin to detect CT or CM, respectively, resulted in recording from CMs five times more often than CTs (see Fig. 1). These estimates are obviously not an exact measure of density, but as our main focus was to record from CT afferents, the higher incidence of CMs in relation to CTs is more likely to be an underestimation, than vice versa. We recently showed that low-threshold C-mechano-afferents are also found in glabrous skin of the human hand, although their presence here appears to be very sparse (34). It is now clear that CT afferents can indeed be found in the distal parts of the limbs such as the hand and lower leg, although the current results suggest that they are considerably sparser in the distal parts of the human body, similar to what has been found in nonhuman primates (35).

### Physiological Properties of CT Afferents across Nerves

CT afferents display several interesting physiological phenomena that are yet to be fully understood. We here resumed the investigation of some of the early observations made on these afferents. Aside from CT afferents’ low mechanical threshold and slow conduction velocity, their vigorous response to brushing and their tuning to brush velocity were similar across the different nerves. Another common feature is afterdischarge, characterized by a vigorous response where a train of impulses follows a natural stimulation. This phenomenon is more prevalent when the stimulus is first applied and therefore often appears just as the unit is being identified. Afterdischarge has also been observed in early recordings from C-nociceptors in the cat (36). In our sample, four out of 20 nociceptor units displayed this feature upon mechanical threshold identification. None of the C-nociceptors showed continued afterdischarge as the stimulus was reapplied. In CT afferents, this was not always the case. Our example (Fig. 2*C*) shows a CT unit that continuously afterdischarges in response to brushing repeated at slow and intermediate velocity. Where ongoing spontaneous activity is considered a sign of pathology in nociceptors (37), there is no indication to date that continuous afterdischarge is a sign of pathology in CT afferents. Delayed acceleration is more unusual and can be reproduced in the units that display this feature (4). Besides the peroneal nerve unit that displayed delayed acceleration in our example, we found this phenomenon in two units tested in the radial and antebrachial nerves. Neither afterdischarge nor delayed acceleration appears to have any clear perceptual correlate. However, a perceptual correlate is likely dependent on spatial summation of units displaying these features simultaneously, which has so far been precluded from study in single-unit recordings alone. Recent studies from pig and human skin have also highlighted that CT afferents have lower thresholds to slowly depolarizing electrical stimuli than C-nociceptors do (28). This may be a useful characteristic to take into account for future design of microneurography studies.

### CT Afferent Response to Menthol

We continued our explorations to include the cooling agent menthol. Animal studies suggested that CLTMs respond to rapid cooling (38–40). Microneurography studies in humans have shown that some CTs respond with a short burst of spikes to cooling (10) but lack a response to heat (4, 10). In addition, the responses of CT afferents to mechanical stimuli can be modified by temperature. Findings from microneurography suggest that the responses of CT afferents are optimal at skin temperature of ∼32°C (18). Furthermore, CT afferent firing is modified by temperature such that touch above skin temperature decreases their firing and cool touch lowers their firing but often produces a longer lasting firing at low frequency, i.e., afterdischarges (16). Most studies on cooling have included a light mechanical impact on the receptive field. The mechanism underlying this combination between mechanical and cool stimulation as well as the response to pure cooling is unknown. It was therefore of great interest to find out whether the cooling agent menthol, that is dependent on the thermosensitive cation channel TRPM8, acts on C-tactile afferents. In the eight units where menthol was topically applied, none was directly activated by menthol. In a subset of units, we also did repeated tests of brushing at different velocities before and after menthol application. Again, we found no indication that the firing rate was modulated by menthol at the time of cooling percept. That menthol does not directly act on CT afferents is consistent with data from rodents suggesting that TRPM8 is not expressed in CLTMs (19). However, recent analysis of gene expression in human dorsal root ganglia suggests that TRPM8 is expressed in a group of touch-sensing cells (41). Although many similarities between mouse and human sensory gene expression have been found, there are clearly discrepancies where TRPM8-sensing neurons are of particular interest.

### CT Afferent Response to Histamine

Next, we initiated CT afferents’ response to histamine, a well-known pruritogen. Although there are various substances that induce itch, histamine is one of the best-known endogenous substances. Histamine acts directly on a prurigenic class of primary sensory neurons containing calcitonin gene-related peptide- and substance P (27, 42). Histamine-induced itch is primarily mediated by mechanoinsensitive C-fibers (MIA) that are also sensitive to heat or capsaicin. However, histamine can also signal through TRPV1, and in primates, it activates mechanosensitive sensory neurons such as A-fiber mechanoheat (AMH) and C-fiber mechanoheat (CMH), although to a weaker extent than MIA (43). None of the CT units in our sample could be directly activated by histamine iontophoresis. Our results are consistent with one of the earliest reports on the physiological properties of low-threshold C-afferents in the cat where histamine did not provoke a consistent response at concentrations of 30% in saline scratched into the skin (38). Because cooling has been reported to modulate the sensitivity of CT afferents to mechanical stimuli, we also tested whether histamine had a similar effect on response to brushing in a subset of these units. Brushing the receptive field after histamine iontophoresis had no clear effect on brushing sensitivity. However, it should be noted that the sample tested with brushing after histamine is low, and the results should therefore not be interpreted as final. The accumulated knowledge from our studies on humans and other species suggests that CT afferents do not play a role in histaminergic itch. However, many questions remain regarding their chemical responsiveness. For example, injection of histamine is more effective to elicit dose-dependent responses in nociceptors than iontophoresis (44). Thus, method of delivery is worth considering for future exploration of CT afferent pruritic sensitivity. The possibility also remains that CT afferents play a role in touch-evoked itch, alloknesis, which is yet to be addressed in detail.

### Low-Threshold C-Mechanoreceptors from Mouse to Human

Molecular visualization of the apparent CT afferent mouse homologue has shown that CT afferents are present in all the nerves supplying the hairy skin (45). Combined with ours and previous recordings from nerves innervating the skin of the face, thigh, and palm (9, 10, 15, 34), this suggests that CT afferents are ubiquitous across human hairy skin, although very sparse in distal parts of the body. Future psychophysical studies should consider this sparsity of CT afferents in distal areas. Recent sequencing studies suggest that human and mouse dorsal root ganglia transcription factors are similar (46) and that there are shared molecular features between mouse and nonhuman primates (47). Although the possibility that there are important differences between the species should be considered, the data from mouse and humans all point in the direction that CT afferents in human and CLTMRs in mice to a large extent exhibit similar physiological properties. As mentioned earlier, functional properties of TRPM8-sensing neurons are of particular interest, as this is where mouse and human sensory gene expression clearly have some discrepancies (41). We here add to the growing body of research including the tuning to brush velocity (1), the combined effects of temperature and mechanical stimulation (16, 18), sensitivity to electrical stimulation (28), and the lack of activation by chemical agents alone in animal models (38). The research from several species suggests that CTs constitute a unique population of cutaneous mechanosensory afferents with highly specialized mechanisms optimal for transducing features of the gentle caressing stimulation seen in affiliative behaviors. However, research from both humans and rodents suggests that integration between the coding properties of several types of afferents is necessary for the percept of, for example, mechanical allodynia in humans (48) and warm temperature in mice (49). The precise contribution of other fiber types for the coding of affective hedonic touch is not known but clearly also depends on Aβ afferents for conscious sensation (2), as well as being heavily modulated by factors such as context and homeostatic state (50).

## GRANTS

This work was supported by Åke Wiberg Foundation (to L. Löken) and by Swedish Research Council 2017-01717 and Västra Götaland Region ALFGBG-725751 (to J. Wessberg).

## DISCLOSURES

No conflicts of interest, financial or otherwise, are declared by the authors.

## AUTHOR CONTRIBUTIONS

L.S.L., H.B.W., H.O., F.M., and J.W. conceived and designed research; L.S.L., H.B.W., and J.W. performed experiments; L.S.L. analyzed data; L.S.L., H.B.W., H.O., F.M., and J.W. interpreted results of experiments; L.S.L. prepared figures; L.S.L. drafted manuscript; L.S.L., H.B.W., H.O., F.M., and J.W. edited and revised manuscript; L.S.L., H.B.W., H.O., F.M., and J.W. approved final version of manuscript.

## APPENDIX

Figure A1 shows an example of afterdischarge in a C-unit with high mechanical threshold (3 g) when stimulated with a sharp mechanical probe. Afterdischarge, where firing continues for several seconds after the stimulus is removed, is often observed in CT afferents. We found that this phenomena also clearly occurs in C afferents with high mechanical threshold.
